# Primary health care workers’ understanding and skills related to cervical cancer prevention in Sango PHC centre in south-western Nigeria: a qualitative study

**DOI:** 10.1017/S1463423619000215

**Published:** 2019-07-01

**Authors:** Anthonia O. C. Onyenwenyi, Gugu Gladness Mchunu

**Affiliations:** 1 School of Nursing and Public Health, University of KwaZulu-Natal, Desmond Clarence Building, 5the Floor, Durban, South Africa; 2 Community Health Officers’ Training School, Lagos University Teaching Hospital, Idi Araba, Lagos, Nigeria

**Keywords:** cervical cancer screening, primary health care workers, training, understanding and skills, visual inspection with acetic acid

## Abstract

**Aim:**

The study explored the knowledge and service delivery skills of primary health care (PHC) workers to conduct cervical cancer screening programmes in Sango primary health centre in Sango town, Ado-Odo Ota, Ogun State in Nigeria.

**Background:**

Cervical cancer is the second most common cancer affecting women. The prevention and control services in Nigeria are provided mainly at post-primary health facilities. Authorities have advocated the integration of cervical cancer prevention into reproductive health services provided at PHC centres. The service delivery capabilities of PHC workers are critical for successful implementation of screening programmes.

**Method:**

Exploratory qualitative research design was used. Data were collected among 10 PHC workers who were purposively sampled at Sango PHC. Semi-structured interview guide with broad items and a checklist were used to assess participants’ cervical cancer screening knowledge and service delivery skills using visual inspection screening methods. Data were analysed thematically and triangulated.

**Findings:**

A range of roles were represented in the interviews of the health care workers at the PHC studied. They had poor knowledge and skills about cervical cancer screening using visual inspection with acetic acid and visual inspection with Lugol’s iodine. Study participants perceived nurses as most equipped PHC workers to conduct screening at PHC level, followed by the community health officers. Participants reported no cervical cancer services at the centre and community. The findings provided useful insight that guided the training of primary health workers and the development of a community-based cervical cancer screening model for women in rural communities.

**Conclusion:**

Nurses and other PHC workers should be trained on visual inspection screening method. This low-cost but effective methodology could be incorporated into their training curricula as a strategy for scaling up cervical cancer prevention programmes across Nigeria.

## Background

Cervical cancer which is caused by the human papilloma virus (HPV) (McGraw and Ferrante, 2014; Finocchario-kessler *et al*., 2016) is the second most common gynaecological cancer (Ferlay *et al*., 2014 cited in GLOBACAN, 2012; Fatungase *et al*., 2013; Ehiemere *et al*., 2015). It is the leading cause of cancer morbidity and mortality in women in Sub-Saharan Africa (SSA). Its prevalence in the region has been described as endemic (Ezechi *et al*., 2013), with 681,000 new cases and 512,400 deaths recorded in 2008. Over 80% of worldwide cervical cancer deaths occurred in low- and middle-income countries (LMIC) where there are no screening services (World Health Organization International Agency for Research on Cancer, GLOBOCAN, 2018; WHO, 2012; McGraw and Ferrante, 2014). The risk factors of cervical cancer among African women include polygamous marriages with associated multiple sexual partners, high parity (more than three children) and giving birth at young age of 17 years, low socio-economic status of women, poor hygiene practices and being HIV-positive or harbouring other immune-compromised statuses (Anorlu, 2008; World Cancer Research Fund/American Institute for Cancer Research, 2014; WHO, 2014). The use of birth control pills for more than five years was reported to be also a risk factor (GLOBOCAN, 2008; Balogun *et al*., 2012; Fatungase *et al*., 2013; WHO, 2014).

Cervical cancer-related mortality can be significantly reduced if women have access to screening services (GLOBACAN, 2008; Ferlay *et al*., 2010; Ebu *et al*., 2015). However, most rural women present with advanced disease stages, due to lack of awareness (Ebu *et al*, 2015); absence of organised screening programmes (Sankaranarayanan, 2014); patients’ delay in seeking health care and delay by health care providers in referring patients to tertiary care facilities (Anorlu *et al*., 2004).

Screening is an effective way to prevent mortality from cervical cancer given the long latency and recognisable pre-cancerous lesions in the disease evolution. It can be treated safely and inexpensively with early detection (Alujuwaihel *et al*., 2013; Ehiemere *et al*., 2015). However, the cytology-based screening method is inaccessible in many LMICs because of costs (Igwilo *et al*., 2012; Sankaranarayanan, 2014; Modibbo et al., 2016), inadequate service infrastructure, lack of skilled manpower and mal-distribution of available human resources (Denny *et al*., 2006).

To redress the current situation in LMICs countries like Ghana, South Africa and Nigeria, low-cost visual inspection with acetic acid (VIA) screening methods have been implemented with varying success rates (WHO, 2009; Igwilo *et al*., 2012; Adefuyea, *et al*., 2013; Poli *et al*., 2015). A comparative study conducted by Albert *et al*. (2012) on VIA and Pap-smear showed that VIA was as sensitive as Pap-smear with a comparable specificity of 94.4–100% and an accuracy of 98.6–99.4%. Similarly, a WHO’s demonstration project showed that is an equally sensitive screening method compared to cytology-based screening (WHO, 2012; Olusegun *et al*., 2016). is cost-effective, cheap (Omole-Ohonsi *et al*., 2013), with a possibility of immediate treatment of identified cases and can be implemented by personnel in primary health care (centres (Poli *et al*., 2015).

Primary health workers are the main drivers of population-oriented health education programmes (Ibama and Dennis 2016). In Nigeria, they facilitated improvements in service utilisation; in fact, the 80% immunisation coverage in the country for vaccine-preventable diseases reported in 2013 was attributed to the role of PHC workers (Ibama and Dennis 2016). The PHC workers provide care closest to community members; they mobilise and empower communities for health actions, thus promoting equity and ensuring accessible health care (Federal Ministry of Health; National Primary Health Care Development Agency, 2012). Therefore, for PHC workers to be effective in providing preventive care for cervical cancer, they require capacity enhancement to assure quality service delivery and better outcomes (Eke *et al*., 2012; Can *et al*., 2014; Catarino *et al*., 2015; Makinde *et al*., 2016).

This study explored the knowledge and service delivery skills of PHC workers to provide cervical cancer screening using visual inspection methods at Sango PHC in Ado-Odo Ota, of Ogun State, Nigeria. Specifically, the research described the demographics of service personnel at Sango PHC and explored their understanding and cervical cancer screening skills using VIA and visual inspection with Lugol’s iodine (VILI) at facility and community levels. We elicited their opinions on how cervical cancer screening services were implemented in the PHC, and which PHC workers are best equipped to provide cervical cancer screening.

## Method

### Study site

The study was conducted in Ogun State in south-western Nigeria. The population of Ogun State was 3,728,098 (National Population Commission, 2006). Administratively, the state is composed of 20 local government areas (LGAs), with Ado-Odo Ota as second-largest LGA with a population of 527,242 (261,532 males and 265,716 females (Nigerian Demographic Profile, 2014). The LGA has 16 political wards, including Sango ward which is in the suburban area of Ado-Odo Ota LGA (Ado Odo/Ota local Government, 2014:13)

### Study facility

The study facility was Sango PHC located in Sango ward. The PHC serves over 140,000 people with an average of 2000 woman seen on monthly basis (facility report, 2016). It was selected purposively because it is strategically located in Sango Ado-Ado Ota LGA of Ogun State, serving suburban as well as rural areas including 14 catchment communities. The PHC was also selected based on its identifiable PHC structure, such as the ward development committee (WDC). Its host village is peaceful with no history of communal conflicts within the preceding two years. The head of Sango PHC oversees the activities of four other health facilities in the ward (two PHCs and two health posts). The latter facilities refer some cases to Sango PHC while staff are rotated among them. Students of community health officers (CHOs) and community health extension workers (CHEWs) training programme of the Lagos University Teaching Hospital and schools of health technology in Ogun State undergo community posting at the facility. Sango PHC also implements several public health projects such as HIV/AIDS, malaria and TB control.

#### Research design, recruitment and participants

This exploratory qualitative study of PHC workers’ perspectives about cervical cancer screening was a component of a larger mixed-method action research project by the author team. The study entailed the development of a community-based cervical cancer screening model for use by rural women. Qualitative data were collected through face-to-face in-depth interviews. Ten out of the 11 workers at the Sango PHC were purposively sampled for the research. Purposive sampling was useful to identify subjects for in-depth investigation that enabled the researcher to gain deeper understanding of the issue of enquiry (Braun and Clarke, 2006). The participants were (i) PHC workers deployed to Sango PHC at the time of the research, (ii) people who had previously participated in community-based programmes such as HIV/AIDS; (iii) people who had worked in two other PHCs and two health posts coordinated by Sango PHC facility manager, and (iv) permanent staff at Sango PHC. Three nurse/midwives, two pharmacy technicians, three CHOs, one CHEW and one laboratory technicians were recruited. The researcher approached them individually and explained the aim of the study and the relevance of their participation. Consenting participants signed informed consent form and were interviewed at a time and place convenient for them within the PHC (see the Supplementary material).

### Study instruments

Qualitative data were collected using pilot-tested interviewer-administered guide which comprised broad items and a checklist to assess participants’ cervical cancer screening knowledge and skills. The questions enabled the researcher to explore and probe the participants to obtain data which addressed the research questions (Pattons, 1990). The interview items elicited information on participants’ demographics, knowledge about cervical cancer, training experiences, skills in using VIA/VILI screening techniques, opinion about which PHC workers were most equipped to participate in the screening activities and how cervical cancer screening services were implemented at Sango PHC.

### Data collection

The data collection process lasted for 16 weeks (13th July to 30th November 2016). The interviews were conducted by the principal investigator (PI) in English language using the interview guide. All interview sessions were audio-recorded and later transcribed verbatim. Confidentiality of information and anonymity of participants were maintained, and participants were assured of this. Data saturation was monitored and reached at the eighth interviewee – when new data repeated what was expressed in the previous data (Saunders *et al*., 2018) and depth of information had addressed the research questions. Two additional participants were interviewed to ensure and confirm that saturation was reached. Guest *et al*. (2006) had proposed that data saturation was the standard by which samples’ size was decided in qualitative health science research such as the current study. Additional data were collected via observations and field notes by the researcher. The observation focused on general organisational environment at the PHC, staff approaches to work, types of services offered in the different units and service days, roles and skills of different cadres of the workers and management of the PHC.

### Data analysis and management

Data were analysed using a thematic approach, and followed the steps outlined by Braun and Clarke (2006): (i) All interviews were audio-recorded, transcribed verbatim and validated by checking back transcripts against original audio recordings for accuracy; (ii) Familiarisation with the data by the PI was achieved through active repeated reading of transcripts and repeated listening to the recorded tapes to arrive at total immersion (Pattons, 1990); (iii) The transcripts were reviewed to identify the themes, patterns and categories. In a three-column tabular format, questions asked were set in the left column, participant responses in the middle column and emerging thematic patterns in the right column. All the participants’ responses to each question were collated; similar and important responses were highlighted in the same shade for ease of identification. The analysis was guided by the research questions, and prevalence was counted at the level of data items by noting the number of participants who mentioned a theme across the data. (iv) This was followed by reviewing and grouping of highlighted related quotes that emerged from the data into themes and sub-themes. Using iterative process and constant comparison, the themes were then collapsed into separate themes. (v) Defining and naming of themes: this involved identifying what was interesting in terms of answering the research questions, considering the relationships among the themes which were presented in tabular form from which report of analysis was generated. Verbatim quotes from participants were used to support each theme. Thereafter, findings were interpreted relating them to similar studies and implication for best practices. Data were reported in broad terms such as most, many, some, few based on the qualitative nature of this study.

#### Triangulation of data

Interviews are most powerful tools for gaining an understanding and exploring topics in depth (Carter *et al*., 2014). The responses from the different categories of the PHC workers including nurses, CHOs, CHEWs, pharmacists, laboratory scientist were triangulated with information from observations and the researcher’s field checklist notes which provided multiple perspectives and ensured robustness and thus validated the data (Denzin, 1978; Carter *et al*., 2014). For instance, observations on participants’ skills in speculum examination were triangulated with the interview data.

#### Trustworthiness, transferability and dependability

Guba’s four criteria for trustworthiness of data, as cited by Shenton (2004), were applied. Credibility was enhanced by developing an earlier familiarity with the culture of the participants before the process of data collection commenced (Lincoln and Guba, 1985). Data collection commenced after the (PI) had worked at the centre for 12 weeks and established relationship with the personnel of Sango . Interview sessions were cross-checked with the understanding of the data by the study participants, by playing back the recorded data to them for corrections. In addition, members of the research team (the supervisor, one nurse/midwife and CHO) reviewed the data to ensure that results were consistent. Detailed description of the research process, its content and participants, the meaning and intentions of the various processes and the study’s conceptual developments were provided (Shenton, 2004), with the aid of a fieldwork journal kept by the researcher throughout the study. Confirmability was ensured through audio-taping of interviews so that participants’ responses were well documented and can be referred to at any time to confirm what they said.

## Findings

### Description of study participants

The mean age of participants was 40.1, and age range was 26–56 years. Of the 10 participants, 8 were women and 2 were men. The highest academic qualification of the participants was bachelor’s degree in nursing (2), while four had a higher national diploma in public health nursing and national diploma in community health (see Table 1).

**Table 1.** The participants’ demographics

Identifier	Gender	Age	Marital status	Religion	Highest academic qualification	Professional designation/role	Tribe
PHC^1^	F	54	Married	Christian	B.Sc. nursing degree	Registered nurse midwife/ward focal person	Yoruba
PHC^2^	F	35	Married	Christian	B.Sc. nursing degree	Public health nurse/community health officer	Yoruba
PHC^3^	F	41	Married	Christian	Higher national diploma in community health	CHO	Yoruba
PHC^4^	F	56	Married	Christian	Higher national diploma in public health nursing	Public health nurse	Yoruba
PHC^5^	F	39	Married	Christian	Diploma in laboratory technician	Medical laboratory technician	Yoruba
PHC^6^	M	46	Married	Christian	Diploma in pharmacy technician	Chief pharmacy technician	Yoruba
PHC^7^	F	33	Married	Islam	Diploma in pharmacy technician	Pharmacy technician	Yoruba
PHC^8^	M	26	Single	Christian	Diploma in health technician	Health assistant	Yoruba
PHC^9^	F	32	Married	Islam	Higher national diploma in community health	CHO	Yoruba
PHC^10^	F	39	Married	Christian	Ordinary national diploma	Community health extension worker(CHEW)	Yoruba

Six broad themes emerged that described the situation regarding the provision of cervical cancer screening services at Sango PHC. These were cervical cancer – an incurable disease of unknown cause, cervical cancer treatment issues, preventing cervical cancer, VIA/VILI experiences, personnel for VIA/VILI screening and barriers to VIA/VILI screening services at PHC.

### Cervical cancer – an incurable disease of unknown cause

Cervical cancer was described by the PHC workers in various ways that indicated their poor understanding of disease causation, recognition, risk factors, characterisation and treatment. Many of the participants described cervical cancer as a ‘disease of unknown cause’, some described it as an ‘incurable disease’, and others acknowledged not knowing the cause. Some of the participants cited risk factors as the cause of the disease; others attributed cervical cancer to complications of poorly treated sexually transmitted infections (STIs) while some perceived it as a disease resulting from ingestion of local herbs. The poor knowledge by the participants was reflected in the typical responses about the description of cervical cancer disease.

“…*Cervical cancer is a* “*disease of unknown cause*” (PHC^1^, nurse/midwife).

“*I heard that it’s not curable…. Don’t know the cause of disease… even though I have heard about it. …I have no idea*” (PHC^7^, pharmacy technician).

“*It is a disease caused by using local medication “Agbo” (traditional herbs)”* (PHC^**8**^, health assistant).

On how cervical cancer can spread and who can be affected, a few described it as a viral disease of cell multiplication that is transmitted sexually, a male-transmitted disease. Many participants enumerated several behaviours that promote acquisition of HPV which causes cervical cancer. The risk factors include being a woman of reproductive age, having poor personal hygiene, having multiple sex partners, using an intrauterine contraceptive device and using certain cosmetics.

“*It is a disease of cell multiplication … viral, sexually transmitted disease, male transmitted disease”* (PHC^**2**^ PHN/CHO).

“*It is the cancer that affects the… vaginal area of the female*” [PHC^**9**^, CHO].

Another participant specified the categories of women the disease affects the most.

“…*The disease affects women of child-bearing age. Women with poor personal hygiene, women having many sex partners or have long standing infections that are not treated and women who used cosmetics that are not prescribed by professionals”* (PHC^**3**^, CHO).

### Cervical cancer treatment issues

Participants demonstrated inadequate knowledge about available treatment options for cervical cancer. Some acknowledged knowing nothing about the treatment. Few identified the use of chemotherapy and radiation. One mentioned periodic and proper screening as being important treatment to ascertain the stage of the disease.

“…*Periodic and proper cervical screening to detect any grade of cervical infection is an important treatment”* (PHC^**4**^, PHN).

Other responses:

“…*. The understanding is that screening should be performed bi-annually, at least one should encourage an adult … an average woman to go for test at least bi-annually, or twice a year, or when patients present related symptoms to know her status*” (PHC^**1**^, nurse/midwife).

“…*I don’t know much about treatment but refer patient to gynaecologist*” (PHC^**10**^, CHEW).

### Preventing cervical cancer

Three sub-themes emerged that described the preventive measures for cervical cancer at the PHC studied: HPV vaccination, early diagnosis of cervical cancer disease and health education and counselling.

#### HPV vaccination

Knowledge about the role of HPV vaccination in cervical cancer prevention was uncommon among the PHC workers interviewed. Only a participant mentioned the use of vaccination as a preventive measure. She understood the vaccine is administered prophylactically to women and young girls, but she was discouraged by unavailability of the vaccine at the PHC. According to her:

“*I have heard about the vaccine which is not available at the PHC but exists at the secondary and tertiary levels of care. I have not taken nor given to my children”* (PHC^**9**^, CHO).

### Recognising cervical cancer

Many participants enumerated recognisable signs and symptoms for early detection of cervical cancer which included pain, vaginal bleeding and offensive discharge. A participant categorised symptoms into early and later symptoms, explaining that the disease has no symptoms in the early stage. Pain was cited as a major early presentation of the disease which affected different parts of the body and physiological processes such as micturition and sexual intercourse. Participants’ response to early signs and symptoms of cervical cancer were depicted in the quotes that follow:

“…*. The symptoms are pain in pelvic area and lower abdomen*” (PHC^**1**^, SNM).

Another characterised the nature of pain, stating:

“…*Early symptoms are painful micturition and painful sexual intercourse*” (PHC^**4**^, PHN).

Another participant added:

“…*Bleeding during intercourse; irregular offensive bleeding/spotting*” (PHC^**10**^, CHEW).

However, one participant maintained that,

“…No symptoms present at the early stage, later it presents as offensive vaginal discharge” (PHC^**2**^, PHN/CHO).

#### Health education and counselling

Empowerment was cited as one of the key strategies for efficacious cervical cancer prevention. Participants reported that owing to the high level of ignorance about cervical cancer, there was a need to promote health education for its control. This was identified as a major role of the PHC workers in scaling up the cervical cancer prevention and screening efforts. The key health education messages identified by participants were prevention of STIs, partner reduction, improved personal hygiene practices, avoidance of consultation with traditional health operators and use of herbs. The health education programmes should be undertaken by trained health workers to educate the community. Participants advocated that education should also focus on men. The quotes that follow are associated with empowerment.

“…*. Firstly, the prevention of cervical cancer starts from health education, because we discovered that many women are ignorant of the cause of cervical cancer. So that’s the first thing I think you need to do to counsel them*” (PHC^**3**^, CHO).

“…*. Cervical cancer prevention is through the health workers’ role; by educating women who are generally ignorant about the disease*” (PHC^**10**^, CHEW).

Another participant identified the relevant health education messages, specifying:

“…*The important health messages are partner reduction; avoid introduction of infection into the vagina during sexual intercourse*” (PHC^1^, SNM).

“.…. *Educating on dangers of going to trado-medical doctors for herbs, using drug to terminate pregnancy*” (PHC,^**10**^ CHEW).

A critical aspect of empowerment for prevention perceived by participants was building capacity of PHC workers to screen women using the VIA method. This proposal was prompted by lessons learned from HIV/AIDS prevention and control programmes in Nigeria, where some PHCs were designated as centres for the prevention of mother-to-child transmission of HIV infection, antiretroviral therapy (ART) centres, with good outcomes: The following response reflected this perception:

“…*We need trained staff to perform the screening in the PHC. Like we have HIV/AIDS focal persons; empower and finance the trained group to implement screening at the PHC*” (PHC^**6**^, pharmacy tech).

#### VIA/VILI experiences

Many participants demonstrated some skill on how to pass vaginal speculum in women and had previously performed the procedure. Similarly, many of the study participants acknowledged the importance of counselling women and obtaining informed consent prior to screening. Only a few had received VIA training previously, but the trained PHC workers could not recall the training content nor describe correctly VIA screening procedure. One of the VIA-trained participants narrated her training experience:

“*Yes, I had training for 2 hours on VIA from church. They first health educated us about VIA. After that they showed us the instruments, and their uses. We were about thirty health workers. The trainers were an NGO, but I did not understand all those things because they just call the health workers in the church to train us* (PHC^3^, CHO).

### Personnel for VIA/VILI screening

Participants’ views about the most equipped PHC workers to participate in cervical cancer screening were assessed. Nurses were perceived as most suitable health workers to conduct screening at PHC level. All categories of nurses were mentioned including nurse/midwife, public health nurses, VIA-trained nurses, nurses who are family planning providers and chief nursing officers. This was followed by the CHOs and CHEWs. Very few participants mentioned doctors. The main reason for not proposing doctors was that no permanent doctor was stationed at the PHC; In the PHC studied, other cadres of health care personnel treated and referred cases to doctors at secondary care level as evidenced here:

“…*We don’t have permanent doctor Emmm I think if possible the doctors, nurses and CHOs*” (PHC^3^, CHO).

“…*.Because there is no doctor here, it’s we [other cadres of health workers] who treat and refer cases*” (PHC^9^, CHEW).

A participant proposed that screening should be performed by female health workers for privacy and the traditional birth attendants (TBAs) should be involved. The rationale was that many rural women will not come to the PHC because they seek care with the TBAs in the community. The following quote depicts rural women’s preference to TBAs:

“…*The TBAs should be involved because some of the women in the community will not come here (referring to Sango PHC). They prefer to go to those TBAs… that’s why am not even happy. The total number of patients that are going to TBA? In-fact, you cannot compare. …If you see their clinic days, the lawyers are going there, bankers are going there, even the medical professionals*” (PHC^**3**^, CHO).

### Barriers to VIA/VILI screening services at PHC

Routine health education and screening services were not provided at Sango PHC as there were no requests for cervical cancer screening from community women they served. The participants identified some service-linked factors that posed as barriers to their ability to provide VIA screening services at the PHC. These barriers were absence of trained manpower, inadequate screening materials and ignorance about the disease among the rural women. Participants’ quotes that follow confirm the current screening situation and associated service-linked barriers.

“…*. We don’t run the test here*” (PHC^**1**^, SNM).

Another confirmed:

“…*. Presently, there is not much awareness about cervical cancer screening, if it exists, people do not know where to go to obtain the screening services*” (PHC ^**9**^, CHO).

“…*We do not provide screening services… due to shortage of personnel, lack of material, because we don’t have the equipment, …. nobody that had been well trained about it*” (PHC^**3**^, CHO).

One of the participants suggested that cervical cancer screening should be incorporated into the existing reproductive health services in the PHC since both services require speculum examination:

“…*.I propose the integration of the cervical screening into family planning services as the procedures involve similar speculum examination*” (PHC^**9**^, CHO).

## Discussion

The majority of the PHC workers interviewed exhibited poor understanding of cervical cancer disease, in terms of its cause, symptoms, associated risk factors and preventive measures. This corroborates the result of similar studies among Haitian health care workers conducted by Zahedi *et al*. (2014); Cameroonian health workers by McCarey *et al*. (2011); secondary health workers in Kaduna (Balarabe *et al*., 2014); female health workers in Ibadan south-western Nigeria (Anyinde and Onugbodun 2003). However, a recent study among health care workers in southern Ethiopia reported that health care workers were knowledgeable about cervical cancer (Dubale *et al*. 2017).

Many participants described cervical cancer to be a sexually transmitted infection and associated it with the highly stigmatised HIV infection. This linkage could discourage screening uptake among rural women. It was also strongly argued that men should be involved in prevention of the disease through health education, empowerment and in encouraging their wives to access screening services. This finding corroborated the reports of Agurto *et al*. (2005), who documented the important roles of men in improving women’s participation and compliance with screening and precancer treatment in Khayelitsh, South Africa.

Participants in our study assumed that recognisable symptoms of cervical cancer were the only reasons for which screening should be undertaken. This assumption could bring about delays in referring women to seek early care. Numerous studies have described cervical cancer as a disease of late presentation, where lack of awareness and delays in case referrals by PHC workers were important causes of women presenting late (Anorlu *et al*., 2004; Ebu *et al*., 2015). Behaviours identified in our study that promoted acquisition of HPV and spread of cervical cancer included the use of family planning drugs and cosmetics use among others. These are consistent with findings in other studies that have identified prolonged use of oral contraceptives as a risk factor (Igwilo *et al*., 2012; Fatungase *et al*., 2013; Makuza *et al*., 2015). However, further research is envisaged on cosmetic usage as a risk factor for cervical cancer.

Poor understanding of causation, disease process and prevention demonstrated by the PHC workers in our study could result in false health information being given to rural communities. Community health workers as preventive health care advocates, community role models and frontline health care providers should be able to provide accurate information that will bring about positive behavioural change. They should be able to succinctly explain the relationship between HPV and cervical cancer to promote understanding, acceptance and uptake of cervical cancer screening by rural women.

Empowerment and health education were proposed as first-line interventions to tackle the high level of ignorance among rural men and women. However, the content proposed for health education did not include issues of HPV vaccination or screening, which should be among the key messages to communicate for behavioural change. Generally, the PHC workers gave evidence of good skills in speculum examination and had previously performed the procedure, unlike respondents in the study by Singh *et al*. (2012) in which 79% of the Sikkimese nursing staff they studied in India reportedly thought that speculum examination was a procedure that needed to be performed by doctors.

The training experience reported by the participants was inadequate in terms of the training site duration and method used for effective VIA screening skills. Blumenthal *et al*. (2005) documented the experiences of Alliance for Cervical Cancer Prevention (ACCP) about the essentials of VIA training stressing that VIA training should be competence-based, combining didactic and hands-on approach and should be done in the clinical settings or at a service delivery programme site. The researchers were not surprised that trained VIA PHC participants could not recall the basic VIA information because training was inadequate.

In the present study, nurses were perceived as most equipped PHC workers to conduct screening at PHC level followed by CHOs. There is large body of evidence from literature showing that screening methods can be successfully implemented by other cadres, if they are trained (WHO, 2009; Shastri *et al*., 2014; Eyitayo, 2015). The examples of PHC workers who could be trained included midwives, laboratory scientist, CHO, CHEW, medical social workers, people entrusted to serve remote and difficult-to-access areas where existing health care services hardly reach. In this study, it was proposed that the TBAs participate in cervical cancer screening because they have high patronage of rural women when compared to the PHC.

Finally, this study confirmed that although doctors have adequate knowledge about cervical cancer (Arulogun and Maxwell, 2012; Eke *et al*., 2012), they do not work permanently at PHCs in Nigeria. Applying the PHC paradigm of resource substitution and task-shifting (Munga *et al.*, 2012; WHO, 2006), the capacity of PHC workers can be developed to implement screening, while women who are screened positive can be referred to the secondary and tertiary levels for appropriate medical evaluation and treatment.

### Study limitations and strength

The findings are not transferable to a larger cohort since it was a regionally based study of 10 PHC workers from a political ward in one local government area of Ogun State in south-western Nigeria.

However, the study provided evidence that informed the training of PHC workers as part of developing a community-based cervical cancer screening model for use by rural women. To minimise the weakness of evidence from one single group, our additional step was to conduct the survey for complementary information that included the views of the rural women who were the users of the proposed model.

### Conclusion and recommendations

Poor knowledge and skills regarding cervical cancer screening were identified among the workers in this study. The multidisciplinary team of these workers who serve as health educators, counsellors and frontline health care personnel can be trained to provide screening services at PHCs.

### Policy and practice implication

The VIA/VILI training programme can be incorporated into the basic curricula for training nurses and other PHC workers as a strategy to scale up cervical cancer prevention and control efforts in resource-limited countries like Nigeria.

## Supplementary material

For supplementary material accompanying this paper visit https://doi.org/10.1017/S1463423619000215. click here to view supplementary material

## References

[ref1] Adefuyea PO , Broutet NJ , de Sanjoséc S and Dennye LA (2013) Trials and projects on cervical cancer and human papillomavirus prevention in Sub-Saharan Africa. *Vaccine* 31S, F53–F59 journal homepage. www.elsevier.com/locate/vaccine 10.1016/j.vaccine.2012.06.070 24331748

[ref2] Agurto I , Arrossi S , White S , Coffey P , Dzuba I , Bingham A , Bradley J and Lewis R (2005) Involving the community in cervical cancer prevention programs. International Journal of Gynaecology and Obstetrics 89, S38–S45. 15823265 10.1016/j.ijgo.2005.01.015

[ref3] Albert SO , Oguntayo OA , Samaila MOA (2012) Comparative study of VIA and Papanicolai (Pap) smear for cervical cancer screening. *Ecancermedicalscience*. Retrieved 5 October 2017. doi: 10.3332/ecancer.2012.262 (http://creativecommons.org/licenses/by/3.0). PMC3408898 22855689

[ref4] Alujuwaihel A , Al-Jarallah A , Al-Busairi H and El-Shazly MK (2013) Awareness of HPV and cervical cancer vaccines among PHC physicians in Kuwait. Greener Journal of Medical Sciences 3, 152–159.

[ref5] Anorlu RI (2008) Cervical cancer: the sub-Saharan African perspective. Reproductive Health Matters 16, 41–49. 19027621 10.1016/S0968-8080(08)32415-X

[ref6] Anorlu RI , Orakwue CO , Oyeneyin L and Abudu OO (2004) Late presentation of patients with cervical cancer to a tertiary hospital in Lagos: what is responsible? European Journal of Gynaecological Oncology 25, 729–732. 15597852

[ref7] Anyinde OA and Onugbodun AO (2003) Knowledge attitude and practices related to prevention of cancer of cervix among female health workers in Ibadan. Journal of Obstetrics and Gynaecology 23, 59–62. 12623487 10.1080/0144361021000043272

[ref8] Arulogun OS and Maxwell OO (2012) Perception and utilization of cervical cancer screening services among female nurses in University College Hospital, Ibadan, Nigeria. Pan African Medical Journal 11, 69. 22655103 PMC3361207

[ref9] Balarabe F , Musa U , Chado MA , Garba SN and Musa HA (2014) Awareness of VIA in cervical cancer screening among nurses in Kaduna state – Nigeria. IOSR Journal of Nursing & Health Science 3, 56–61.

[ref10] Balogun MR , Odukoya OO , Oyediran MA and Ujomu PI (2012) Cervical cancer awareness and preventive practices: a challenge for female urban slum dwellers in Lagos, Nigeria. African Journal of Reproductive Health 16, 75–82. 22783671

[ref11] Blumenthal PD , Lauterbach M , Sellors JW and Sankaranarayanan R (2005) Training for cervical cancer prevention programs in low‐resource settings: Focus on visual inspection with acetic acid and cryotherapy. International Journal of Gynecology & Obstetrics 89, S30–S37. 15823264 10.1016/j.ijgo.2005.01.012

[ref12] Braun V and Clarke V (2006, Jan 1) Using thematic analysis in psychology. Qualitative Research in Psychology 3, 77–101.

[ref13] Can H , Erdem O , Oztekin C , Celik SB , Onde M , Celepkolu T and Ongel K (2014) Are primary health care workers aware of cervical cancer risk? Asian Pacific Journal of Cancer Prevention 15, 6669–6671. 25169506 10.7314/apjcp.2014.15.16.6669

[ref14] Carter N , Bryant-Lukosius D , Di Censo A and Neville AJ (2014) The use of triangulation in qualitative research. Oncology Nursing Forum 41, 545–547. 25158659 10.1188/14.ONF.545-547

[ref15] Catarino R , Vassilakos P , Scaringella S , Undurraga-Malinverno M , Meyer-Hamme U , Ricard-Gauthier D , Matute JC and Petignat P (2015) Smartphone use for cervical cancer screening in low-resource countries: a pilot study conducted in Madagascar. PloS One 10, e0134309. doi: 10.1371/journal.pone.0134309. PMC4519052 26222772

[ref16] **Community Health Practitioners’ Registration Board of Nigeria** (2006) Curriculum for higher diploma in community health. Reproductive Health, Abuja, Nigeria pp. 51–56.

[ref17] Denny L , Quinn M and Sankaranarayanan R (2006, Aug 21) Screening for cervical cancer in developing countries. Vaccine 24, S71–S77. 10.1016/j.vaccine.2006.05.121 16950020

[ref18] Denzin NK (1978) The research act: a theoretical introduction to sociological methods, second edition New York: McGraw-Hill.

[ref19] **Department of information, education and sports Ado Odo/Ota local Government** (2014) Assessment of Ado-Odo/Ota local government. pp. 13–14.

[ref20] Dubale D , Daka D and Wakgari N (2017) Knowledge about cervical cancer screening and its practice among female health care workers in southern Ethiopia: a cross-sectional study. International Journal of Women's Health 9, 365–372. doi: 10.2147/IJWH.S132202. PMC5446960 28579837

[ref21] Ebu NI , Mupepi SC , Siakwa MP and Sampselle CM (2015) Knowledge, practice, and barriers toward cervical cancer screening in Elmina, Southern Ghana. International Journal of Women’s Health 7, 31–39. 10.2147/IJWH.S71797 PMC4284003 25565902

[ref22] Ehiemere IO , Frank MD and Robinson-Bassey GC (2015) Attitude and practice of cervical cancer screening among female health workers in University of Port-Harcourt Teaching Hospital, Rivers State. Journal of Research in Nursing and Midwifery 4, 72–82.

[ref23] Eke NO , Eke CO , Nwosu BO , Akabuike JC , Ezeigwe CO and Okoye SC (2012) Cervical cancer screening by female workers in South East Nigeria. Afrimedic Journal 3, 11–15.

[ref24] Eyitayo L (2015, Dec 8) Primary health care realities, challenges and the way forward. 1st annual primary health care lecture: organised by the National Primary Health Care Development Agency Abuja. Retrieved 4 August 2017 from http://nigeriahealthwatch.com/wp-content/uploads/bsk-pdf-manager/1160_2015_Primary_Health_Care_Presentation_Final,_NPHCDA_1216.pdf

[ref25] Ezechi OC , Gab-Okafor CV , Ostergren PO and Pettersson KO (2013) Willingness and acceptability of cervical cancer screening among HIV positive Nigerian women. BMC Public Health 13, 46. 23327453 10.1186/1471-2458-13-46 PMC3567931

[ref26] Fatungase OK , Olu-Abiodun OO , Idowu-Ajiboye BA and Awosile JO (2013) An assessment of women’s awareness and knowledge about cervical cancer and screening and the barriers to cervical screening in Ogun State, Nigeria. IOSR Journal of Dental and Medical Sciences 10, 52–58.

[ref27] **Federal Ministry of Health; National Primary Health Care Development Agency** (2012) *Minimum standard for primary health care in Nigeria. Department of Planning, Research and Statistics. Ch 5 Primary Health Care Centre. Retrieved 20 September 2018*. doi: 10.1002/ijc.25516

[ref28] Ferlay J , Shin HR , Bray F , Forman D , Mathers C and Parkin DM (2010) Estimates of worldwide burden of cancer in 2008: GlOBOCAN 2008. International Journal of Cancer 127, 2893–2917. 21351269 10.1002/ijc.25516

[ref103] Ferlay J , Soerjomataram I , Ervik M , Dikshit R , Eser S , Mathers C , Rebelo M , Parkin DM , Forman D , and Bray F (2012) Globocan. 2012. Cancer incidence and mortality worldwide: IARC Cancer Base No. 11. Lyon, France: International Agency for Research on Cancer.

[ref29] Finocchario-Kessler S , Wexler C , Maloba M , Mabachi N , Ndikum-Moffor F and Bukusi E (2016) Cervical cancer prevention and treatment research in Africa: a systematic review from a public health perspective. BMC Women’s Health 16, 29. 27259656 10.1186/s12905-016-0306-6 PMC4893293

[ref31] Guest G , Bunce A and Johnson L (2006) How many interviews are enough? An experiment with data saturation and variability. Field Methods 18, 59–82. doi: 10.1177/1525822X05279903.

[ref32] Ibama AS and Dennis P (2016) Role of community health practitioners in national development: the Nigeria situation. International Journal of Clinical Medicine 7, 511–518.

[ref33] Igwilo AI , Igwilo UU , Hassan F , Idanwekhai M , Igbinomwanhia O and Popoola AO (2012) The knowledge, attitude and practice of the prevention of cancer of the cervix in Okada Community. Asian Journal of Medical Sciences 4, 95–98.

[ref35] Lincoln YS and Guba EG (1985) Naturalistic inquiry. Beverly Hills CA: Sage.

[ref36] Makinde B , Akinremi T , Solademi A , Olaoye T , Ogunsanmi O and Dangana J (2016). Cancer of the cervix and cervical screening: current knowledge, attitude and practice of PHC workers in Ikene LGA Ogun State, Nigeria. IOSR, Journal of Nursing and Health Sciences 5, 62–65.

[ref37] Makuza JD , Nsanzimana S , Muhimpundu MA , Pace LE , Ntaganira J and Riedel DJ (2015) Prevalence and risk factors for cervical cancer and pre-cancerous lesions in Rwanda. Pan African Medical Journal 22, doi: 10.11604/pamj.2015.22.26.7116. PMC4662515 26664527

[ref38] McCarey C , Pirek D , Tebeu PM , Boulvain M , Doh AS and Petignat P (2011) Awareness of HPV and cervical cancer prevention among Cameroonian healthcare workers. BMC Womens Health 18;11, 45. doi: 10.1186/1472-6874-11-45. PMC3219551 22008186

[ref39] McGraw SL and Ferrante JM (2014) Update on prevention and screening of cervical cancer. World Journal of Clinical Oncology 5, 744. 25302174 10.5306/wjco.v5.i4.744 PMC4129537

[ref40] Modibbo FI , Dareng E , Bamisaye P , Jedy-Agba E , Adewole A , Oyeneyin L , Olaniyan O and Adebamowo C (2016) Qualitative study of barriers to cervical cancer screening among Nigerian women. BMJ Open 6, e008533. 10.1136/bmjopen-2015-008533 PMC4716205 26754174

[ref41] Munga MA , Kilima SP , Mutalemwa PP , Kisoka WJ and Malecela MN (2012) Experiences, opportunities and challenges of implementing task shifting in underserved remote settings: the case of Kongwa district, central Tanzania. BMC International Health and Human Rights 12, 27–38. 23122296 10.1186/1472-698X-12-27 PMC3503551

[ref100] **National Population Commission** (2006) National Population Census. Abuja, Nigeria: National Population Commission.

[ref43] **Nigeria Demographics Profile** (2014). Retrieved 24 June 2013 from http//www.indexmundi.com/nigeria/demographics_profile

[ref44] Olusegun GE , Oluwaserimi AA , Akinwumi AO , Olusegun BA , Adebayo AA and Dada SA (2016) Reliability of visual inspection after acetic acid staining in screening for cervical pre-malignant lesion among female subjects in a Rural Tertiary Hospital in Nigeria. Cancer Research Journal 4, 1–8. doi: 10.11648/j.crj.20160401.11.

[ref45] Omole-Ohonsi A , Aiyedun TA and Umoru JU (2013) Diagnostic accuracy of VIA compared to Pap smear cytology in detecting premalignant lesions of the cervix. African Journal of Medical and Health Sciences 12, 25.

[ref46] Pattons M (1990) Qualitative evaluation and research methods, second edition Newbury Park CA: Sage.

[ref47] Poli UR , Bidinger PD and Gowrishankar S (2015) Visual inspection with acetic acid (via) screening program: 7 years’ experience in early detection of cervical cancer and pre-cancers in rural South India. Indian Journal of Community Medicine: Official Publication of Indian Association of Preventive & Social Medicine 40, 203. 26170547 10.4103/0970-0218.158873 PMC4478664

[ref48] Sankaranarayanan R (2014) Screening for cervical cancer in low- and middle-income countries Icahn School of Medicine at Mount Sinai. Annals of Global Health 80, 412–417. doi:10.1016/j.aogh.2014.09.014. 25512156

[ref49] Saunders B , Sim J , Kingstone T , Baker S , Waterfield J , Bartlam B , Burroughs H and Jinks C . (2018) Saturation in qualitative research: exploring its conceptualization and operationalization. Quality & Quantity 52(4), 1893–1907. doi:10.1007/s11135-017-0574-8 29937585 PMC5993836

[ref50] Shastri SS , Mittra I , Mishra GA , Gupta S , Dikshit R , Singh S and Badwe RA (2014) Effect of VIA screening by primary health workers: randomized controlled study in Mumbai, India. Journal of the National Cancer Institute 106, dju009. 10.1093/jnci/dju009 PMC3982783 24563518

[ref52] Shenton AK (2004) Strategies for ensuring trustworthiness in qualitative research projects. Education for Information 22, 63–75.

[ref51] Singh E , Seth S , Rani V and Srivastava DK (2012) Awareness of cervical cancer screening among nursing staff in a tertiary institution of rural India. Journal of Gynecologic Oncology 23, 141–146. 22808355 10.3802/jgo.2012.23.3.141 PMC3395008

[ref55] **World Health Organization** (2009) Cervical cancer prevention pilot project in 6 African countries. Antananarivo: Madagascar Blantyre: Malawi Lusaka: Zambia Masaka: Uganda Peramiho/Moshi; Tanzania. Sagamu: Nigeria.

[ref56] **World Health Organization** (2012) Prevention of cervical cancer through screening using visual inspection with acetic acid (VIA) and treatment with cryotherapy.

[ref57] **World Health Organization GLOBOCAN** (2008) Global cancer facts and figure, second edition. Lyon Cedex, France: GLOBACAN.

[ref58] **World Health Organization International Agency for Research on Cancer, GLOBOCAN** (2012) Estimated cancer incidence, mortality and prevalence worldwide in 2012.

[ref59] **World Health Organization (WHO)** (2006) The world health report 2006; working together for health. Geneva: World Health Organization Retrieved 29 September 2018 from http//www.who.int/whr/2006

[ref60] **World Health Organization (WHO)** (2014) International Agency for Research on Cancer In Stewart BW and Wild CP , editors, World cancer report. Lyon, France: International Agency for Research on Cancer.

[ref61] **World Health Organization (WHO), International Agency for Research on Cancer, GLOBOCAN** (2012) Estimated cancer incidence, mortality and prevalence worldwide in 2012.

[ref62] Zahedi L , Sizemore E , Malcolm S , Grossniklaus E and Nwosu O (2014) Knowledge, attitudes and practices regarding cervical cancer and screening among Haitian health care workers. International Journal of Environmental Research and Public Health 11, 11541–11552. 25390794 10.3390/ijerph111111541 PMC4245628

