# Supplementary material for: Primary health care workers’ understanding and skills related to cervical cancer prevention in Sango PHC centre in south-western Nigeria: a qualitative study
Source: Prim Health Care Res Dev. 2019 Jul 1;20:e93. doi: 10.1017/S1463423619000215 (PMC6609971; doi:10.1017/S1463423619000215)
Supplement: Supplementary file 1 [file S1463423619000215sup001.docx]

# Interview schedule; an appendix as supplementary material.

# APPENDIX A: Consent form for individual interviewees

SCHOOL OF NURSING AND PUBLIC HEALTH

Doctor of Philosophy - Ph.D (Nursing)

Researcher:

Supervisor:

Dear Participant,

**Introduction:** My name is ………………. I am a Doctoral student registered at the School of Nursing and Public Health, Howard College Campus, University of KwaZulu-Natal, Durban, South Africa. My supervisor is Professor …………………. of the School of Nursing and Public Health, University of KwaZulu-Natal.

You are invited to participate in this research study titled “Developing a Community-Based cervical cancer Prevention Model for Rural Women in Sango Ota, Ogun State, Nigeria”.

**Purpose of the Study & procedure:** The purpose of the research is to identify how best to provide cervical cancer screening services to rural women in Sango communities. You will be requested to answer question about the implementation of cervical cancer screening. You will also be collaborating with the researchers to find solution to the problem of why women are not accessing cervical screening.

**Study benefit:** I hope that by this study, more light will be thrown on how best to motivate rural women to undertake the cervical screening as a preventive measure. The outcome will help to inform better policies on the prevention of cervical cnacer in Sango and Nigeria at large.

**Voluntary Participation:** Should you accept to take part in the study, you will be requested to participate in two in-depth interview sessions. One session will hold at the beginning and another at the end of the study. The interview sessions will last for about 90 minutes (1hour 30min). The interview will be conducted by the researcher and assistants. The interview will be audio taped, transcribed verbatim and coded. During the interview I will request you to share your experiences concerning your views about factors that can serve as barriers (knowledge, practices, beliefs, socio economic, religious, political, service related factors e.g. where to obtain service etc.) in your experience that is limiting rural women and how we can solve the problem.

**Risk / Harm to participants:** Please be informed that there is NO risk or harm to yourself or others for participating in this study. Should you experience any discomfort during interviewing, you have the right to refuse to respond or to withdraw from the interview process. You are under no obligation to participate in this study.

**Confidentiality:** Confidentiality of information will be maintained always. Your identity will be protected, tapes of interviews and transcribed material will be kept safe always by the researcher. Extracts from your interviews may be incorporated into the thesis, future academic articles, professional conferences and seminars that may emanate from this study, without revealing your identity. Please where I require clarification, further information of interview responses, I will call you to arrange a follow-up interview at your convenience.

**Safety of research materials:** For purposes of assessing the study’s validity, I will keep all relevant documents and artefacts pertaining to the study to enable an independent researcher not part of the study to track and assess links and connections between the raw data and the final report. After five years upon completion of the study and the awarding of the degree, audiotapes will be destroyed.

Should you agree to participate based on having read and understood the nature and conditions of this research study, please sign the designated section below. Should you have concerns about this study please contact details below.

BIOMEDICAL RESEARCH ETHICS ADMINSTARTION

Research Office, Westville Campus,

Govan Nbeki Building,

University of KwaZulu – Natal

Private Bag X 54001, Durban, 4000

KawZulu-Natal South Africa

Tel: 27312602486 – Fax: 27312604609

Email: BREC2ukzn.ac.za

Researchers’ contact

Researchers are:

School of Nursing and Public Health,

Howard College Campus

University of KwaZulu-Natal

Tel: +234(0)8033013629 (Nigeria)

+27(0)749052507 (South Africa)

e-mail: oguoguo2001@yahoo.com

**Contact details of Supervisor:**

Professor in Community Health

School of Nursing and Public Health

Howard College Campus
University of KwaZulu-Natal

Tel: +27 (0)31 2601421

**PLEASE SIGN THE CONSENT SHEET OVER LEAF**

**-----------------------------------------------------------------------------------------------------------**

**PARTICIPANT DECLARATION**

**Consent for qualitative interviews**

**I …………………………………………………………....** (Full names of participant) hereby confirm that I understand the contents of this document and the nature of the research project, and consent to participating in the study. I also grant permission for interviews to be audio taped, and for transcribed interview material to be utilized for research purposes.

# APPENDIX B: Interview schedule for community health workers (CHWs)

**Section A: Fact sheet for in-depth interview**

Time --------------- Date ---------------------- Place/ Venue -----------------------------------------

Phone ---------------------------------------- Emails ----------------------------------------------------

Any condition that may affect interview session: - ----------------------------------------------------

Local Government Area…………………Name of PHC -------------------------------------------

**Section A: Demographic data**

*Provide information on the following:*

1. Marital status: single ( ) married ( ) divorced ( ) widow ( ) widower ( )
2. Age: please write in years ---------------------------------
3. Gender Male ( ) Female ( )
4. Religion: Christianity ( ) Islam ( ),Traditional( ) others specify…………………………
5. Academic qualification: No formal education ( ) primary education ( ) secondary ( ) tertiary ( ) others specify………………………………………-------------------------
6. Professional qualification --------------- Date ------------- Duty station at PHC ------------
7. Tribe: Hausa ( ) Ibo ( ) Yoruba ( ) Others Specify………………………………………

**Section B**

1. Can you describe the common reproductive cancers that women present in the PHC centre? (probe cervical cancer?)

2. What is your understanding about cervical cancer disease? (In terms of disease causation, presentation, screening and treatment)

3. What can you say about how cervical cancer disease is prevented? (Probe: primary secondary and tertiary levels of prevention).

4. What is your understanding of the screening approaches for cervical cancer (probe developed and developing countries like Nigeria or rural communities like your catchment area?)

5. What forms of cervical cancer prevention/screening do you implement in this Sango PHC facility?

6. Describe the category of health workers who are trained to performs cervical cancer screening in your PHC?

7. Describe the forms of training have you had related to cervical cancer screening using Visual Inspection with acetic Acid and Lugos Iodine? (***See checklist below in section C****)*

8. Please tell me any other thing about cervical cancer prevention/ screening programme in this PHC?

**SECTION C (Checklist on VIA/VILI screening skills)** *Please provide information on the following:*

1. Have you received any training on cervical cancer screening skill? Yes ( ) No ( )

a) Which screening method were you trained on ………………………………………….

b) How do you pass vaginal speculum for a woman in this facility? (Probe; steps in speculum examination establishing rapport; privacy, instrument selection; infection control etc )

c) Counselling /health education is good but not necessary before screening a woman.

Yes ( ) No ( )

d) Obtaining patients consent is not necessary before screening a women Yes ( ) No ( )

***Thank you.***
